# Verification of a Novel Minimally Invasive Device for the Isolation of Rare Circulating Tumor Cells (CTC) in Cancer Patients’ Blood

**DOI:** 10.3390/cancers14194753

**Published:** 2022-09-29

**Authors:** Paul Friedrich Geus, Felix Hehnen, Sophia Krakowski, Klaus Lücke, Dave S. B. Hoon, Nikolaj Frost, Ulrich Kertzscher, Gabi Wendt

**Affiliations:** 1 Biofluid Mechanics Laboratory, Institute of Computer-assisted Cardiovascular Medicine, Charité-Universitätsmedizin Berlin, 13353 Berlin, Germany; 2 Invicol GmbH, Müllerstraße 178, 13353 Berlin, Germany; 3 HaimaChek Inc., 2200 Santa Monica Blvd, Santa Monica, CA 90404, USA; 4 Department of Translational Molecular Medicine, Saint John’s Cancer Institute (SJCI), Providence Saint John’s Health Center (SJHC), Santa Monica, CA 90404, USA; 5 Department of Infectious Diseases and Respiratory Medicine, Charité-Universitätsmedizin Berlin, 13353 Berlin, Germany

**Keywords:** circulating tumor cells (CTCs), minimally invasive, liquid biopsy, non-small-cell lung carcinoma, cancer diagnosis, medical device

## Abstract

**Simple Summary:**

Detection of circulating tumor cells (CTCs) in blood can be used to diagnose cancer or monitor treatment response for various cancers. However, these cells are rare in the bloodstream in the early stages of cancers, and it, therefore, remains a technical challenge to isolate them. To overcome the limitations of a blood draw, we introduce a minimally invasive device, called the BMProbe™, for the isolation of CTCs directly from the bloodstream. Thereby a large volume of blood is screened. This study first shows how the geometry of the in vivo BMProbe™ causes improved cell deposition conditions. We then performed a verification of the in vivo device using blood samples from lung cancer patients. The results indicate the functionality of the BMProbe™ to isolate CTCs in blood samples. The future step is to use the BMProbe™ in various types of cancer patients to detect CTCs.

**Abstract:**

Circulating tumor cells (CTCs) exist in low quantities in the bloodstream in the early stages of cancers. It, therefore, remains a technical challenge to isolate them in large enough quantities for a precise diagnosis and downstream analysis. We introduce the BMProbe™, a minimally invasive device that isolates CTCs during a 30-minute incubation in the median cubital vein. The optimized geometry of the device creates flow conditions for improved cell deposition. The CTCs are isolated using antibodies that are bound to the surface of the BMProbe™. In this study, flow experiments using cell culture cells were conducted. They indicate a 31 times greater cell binding efficiency of the BMProbe™ compared to a flat geometry. Further, the functionality of isolating CTCs from patient blood was verified in a small ex vivo study that compared the cell count from seven non-small-cell lung carcinoma (NSCLC) patients compared to nine healthy controls with 10 mL blood samples. The median cell count was 1 in NSCLC patients and 0 in healthy controls. In conclusion, the BMProbe™ is a promising method to isolate CTCs in large quantities directly from the venous bloodstream without removing blood from a patient. The future step is to verify the functionality in vivo.

## 1. Introduction

Circulating tumor cells (CTCs) represent a subset of tumor cells from an existing primary or metastatic tumor in a cancer patient. They are defined as cells that are shed from the primary or metastatic tumors and subsequently circulate through the bloodstream. They reflect the heterogeneity of the primary or metastatic tumors, in that their analysis can provide clinically relevant information on the patient’s metastatic ongoing potential. Therefore, CTCs have the potential to establish themselves as an essential blood biomarker surrogate in the clinical setting [1,2,3].

CTCs can be used for diagnosis, to monitor therapeutic responses, and for prognosis evaluation, as shown, for example by Lozano et al. in metastatic castration-resistant prostate cancer (PCa) patients. The study showed that the CTC count was superior to the established Prostate Specific Antigen biomarker (PSA) in predicting survival [4]. Further studies have shown the great potential of CTCs in breast cancer [5], colorectal cancer [6], and lung cancer [7]. Additional utility of application for CTCs is related to drug resistance testing [8], the detection of minimal residual disease after therapy, disease recurrence, and early diagnosis of cancer [9,10].

However, there are still challenges to overcome to establish CTCs as a routine biomarker for making patient management decisions. The main technical challenge is the very low levels of CTCs in the bloodstream, particularly in early-stage disease. There are around 1–10 CTCs in 1 mL of whole blood in the advance states of metastatic patients and far fewer CTCs in non-metastatic patients [11,12]. This makes it very difficult to detect CTCs, considering that 1 mL of whole blood consists of approximately 7.5 × 10^6^ white blood cells, 7 × 10^9^ red blood cells, and 3.2 × 10^8^ platelets [13]. Due to the low quantities of CTCs, Coumans et al. recommend processing larger volumes of blood [14]. Yet, since CTCs were first observed by Ashworth et al. in 1869 [15], most methods that have been developed for the enrichment of CTCs focus on an ex vivo 7.5–10 mL blood draw. Although various approaches for the detection of CTCs have been developed, Lin et al. conclude that “the sensitivity and specificity of these technologies must still need to be further improved” [16] (p. 16). Most importantly, Yang et al. have questioned whether this small sample volume of blood draw accurately reflects the detection frequency in an adult human patient [17]. Additionally, it has been questioned whether isolating one to five cells from a 10 mL blood draw is truly representative of metastasis. Further, to circumvent the potential problems that occur through collecting and shipping of blood samples, blood is often collected in tubes that causes “cell death and crosslinking of intracellular contents of CTCs that affect functional analysis, and especially single cell CTC characterization or ex vivo expansion of CTCs” [17] (p. 12).

Therefore, in vivo approaches are required that circumvent the limitations and problems associated with a blood draw, and most importantly enhance the sampling probability of detection of CTCs. Two examples of in vivo CTC enrichment methods are the MagWIRE concept [18] and the CE-approved CellCollector^®^ by Gilupi GmbH, Potsdam, Germany [19]. Both approaches are minimally invasive, and they are both inserted into the cubital vein, where they remain between 30 min to one hour. During their incubation period, they isolated epithelial cell adhesion molecule (EpCAM) positive cells using two different antibody-based approaches. For the MagWIRE, magnetic particles coated with antibodies (Ab) are injected into the bloodstream. Then, a magnetic wire, called MagWIRE, is inserted into the patient’s cubital vein. This wire then isolates the target cells based on magnetic forces. The CellCollector^®^ isolates the cells based on an antibody-antigen interaction. This means the binding capabilities of the CellCollector^®^ are far more sensitive to the flow conditions around the surface of the probe than with the MagWIRE approach. Theoretically, these in vivo applications should isolate a much larger number of CTCs compared to ex vivo alternatives. While the MagWIRE has not yet been tested in humans, there are publications showing that the in vivo cell binding of the CellCollector^®^ is not as superior to ex vivo methods as expected [20,21]. Subsequently, Dizdar et al. analyzed the flow surrounding the surface of the CellCollector^®^ using an in silico analysis. They calculated that the screened blood volume is in the region of 0.33–18 mL per half hour. They considered blood as screened if it passed the probe at a maximum distance of 50 µm [21]. This shows, that for a successful in vivo isolation of CTC in large quantities, a geometry that allows for improved cell deposition conditions is required.

This report introduces a novel in vivo device, called the BMProbe™. With experimental results, it will be shown that its geometry creates flow conditions in the vein that facilitate improved cell deposition. Whereby, the functionality will be validated through ex vivo experiments using blood from lung cancer patients.

## 2. Materials and Methods

### 2.1. Flow Optimized Geometry

When developing the geometry of an invasive cell collection device, two parameters must be considered: The screened blood volume and the area of the device with a negative normal flow. This is stressed by Hehnen et al. who determined that cell deposition is most likely to occur in areas where the flow is normal to the surface of the wall (negative wall normal rate) [22]. It is further essential to avoid a laminar flow profile around the geometry of the inserted cell collection probe. In a laminar flow profile, the cell movement is based on the streamlines around the probe and thus the cells do not collide with the surface of the probe. In these cases, only diffusion causes cell transport in the direction of the probe surface [23].

**Figure 1:**
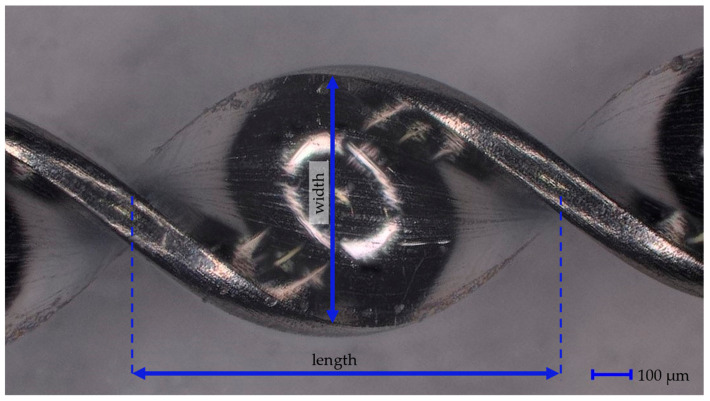
Magnified view of one winding. Captured using 3D-Depth imaging with a digital microscope (VHX-970F, Keyence, Japan) at 100× magnification. Each winding has a length of 1105 μm (±20 μm), a width of 800 μm (±80 μm), and a thickness of 150 μm (±15 μm).

The BMProbe™ was therefore designed to disrupt the blood flow and cause it to “stumble” and collide onto the surface of the probe. The windings of the probe cause areas with negative normal flow, which causes improved cell attachment conditions. The foundation of the device is a medical grade stainless steel wire (Ulbrich, North Haven, CT, USA) with a rectangular cross-section with dimensions of 0.80 mm × 0.15 mm. In total, the wire has a length of 160 mm. Over a length of 40 mm, it is coated with a photoactive polymer (trademarked procedure) to which Abs are covalently bound through UV-cross-linking. This 40 mm section is called the functionalized part. It consists of the tip, 32 windings, and the transition from spiralized to a flat wire. Each winding has a length of 1105 μm (±20 μm) and a width of 800 μm (±15 μm). The twisting of the wire is performed in-house using a specifically designed twisting machine. For this study, spot checks were performed using a digital microscope (VHX-970F, Keyence, Osaka, Japan) at 100× magnification. Of the 50 performed measurements regarding the dimensions of the windings, all dimensions were within the limit of agreement. A magnified image showing the spiral structure can be seen in Figure 1.

**Figure 2:**
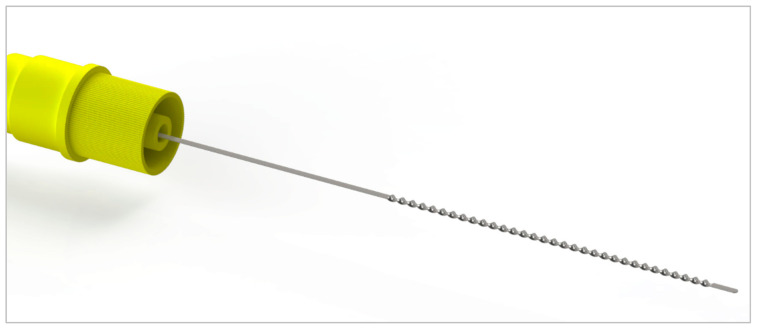
BMProbe is a medical-grade stainless steel wire with 32 windings. The windings are coated with a photoactive polymer and IgG-Ab that isolate specific rare cells from the venous bloodstream. The yellow IN-connector is screwed onto an indwelling cannula (18G × 1¼″) that is inserted into the cubital vein.

The coating of the functionalized part enables the application of any IgG Ab onto the surface of the probe. This allows for the application of the BMProbe™ in different diseases. For an application in oncology, it can be coated, for example, with anti-EpCAM or anti-Vimentin Ab. By coating with other non-oncology related antibodies such as anti-CD105 or anti-CD146 Ab, it can be applied in cardiology to diagnose cardiac issues such as heart failure or arterial hypertension. The different layers (polymer and Ab) are applied to the wire through crosslinking steps that are performed after the application of each layer. The geometry of the probe is shown in Figure 2.

The wire is inserted into the median cubital vein through an indwelling cannula (18G × 1 ¼″), where it is left for a 30-min in vivo incubation time. Then, it is withdrawn and washed gently with a Phosphate-buffered Saline (PBS) (ROTI^®^Cell PBS, Carl Roth GmbH + Co. KG, Karlsruhe, Germany) solution that has a pH value between 7.3 and 7.5. This step removes blood droplets from the surface of the BMProbe™. If the BMProbe™ is to be analyzed at a later time-point, or the viability of cells is not necessary, the probe is fixed using an acetone solution at 4 °C to avoid cellular decomposition. The cells bound to the surface of the BMProbe™ can be counted using immunofluorescence imaging, see Section 2.4. Subsequently, molecular analysis is possible to perform an analysis of the DNA and RNA content of the bound cells.

### 2.2. Flow System

**Figure 3:**
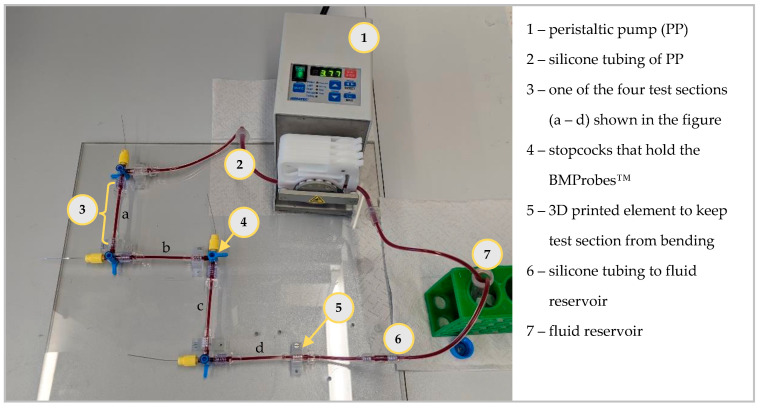
The developed flow system recreates the anatomical and physiological conditions of the median cubital vein. The PP pumps the blood from the fluid reservoir to the test sections using the silicon tubing of the PP. The number of test sections in the flow system can be increased. The 3D printed elements improve reproducibility by ensuring a straight test section.

The geometry of the BMProbe™ was determined using an experimental model analysis. For this analysis, a similar flow system to the one shown by Hehnen et al. was used. The flow system, see Figure 3, was developed to depict the median cubital vein in diameter and flow velocity. It consists of a peristaltic pump (PP) (Ismatec ISM597D, Glattbrugg, Switzerland), silicone tubes (inner diameter 3 mm, outer diameter 5 mm) for the PP, a specifically designed test section (see [22] for detailed description) that fits the functional part of the BMProbe™, stopcocks to connect the components, and a fluid reservoir and further silicone tubes that connect the test section to the fluid reservoir (inner diameter 2 mm and outer diameter 4 mm). There are different versions of test sections. They exist with a diameter of 2 mm, 3 mm, and 4 mm. These diameters are based on literature values of the median cubital vein diameter [18,24,25]. The BMProbe™ is inserted into the flow system through IN-connectors. Unlike in the procedure for in vivo application, where the BMProbe™ is inserted at an angle to the flow, it is positioned horizontally in the middle of the test section. This setup was chosen to improve the reproducibility of the results, as different insertion angles would influence the fluid-surface interaction. To avoid bending of the test sections, mounting brackets were 3D printed (Formlabs, Form 3, Somerville, MA, USA) using the material Clear V4 (Formlabs, USA). The mounting brackets were screwed onto a Plexiglas pane so that the test sections do not bend and are positioned vertical to one another. It is possible to arbitrarily increase the number of probes that are included during each experiment by increasing the number of test sections. This enables the analysis of multiple and/or different probes in the same fluid.

During experiments, the flow system is filled with either a dextran solution (Carl Roth GmbH + Co. KG, Karlsruhe, Germany), which mimics the non-Newtonian flow properties of blood, containing cell culture cells, or patient blood. When inserting patient blood into the flow system, it must be blocked using 3% bovine serum albumin (BSA)/PBS for 30 min to avoid coagulation and an increase in unspecific binding. The flow system can also be heated to human body temperature of 37 °C by placing it in a heated water bath. However, experiments showed that this had no influence on the binding efficacy of the BMProbe™. The probes are inserted one after the other. Starting with the probes closest to the PP, when starting the experiment. When the BMProbes™ are withdrawn, the probe farthest from the PP is withdrawn first. This procedure avoids contact between the BMProbes™ and air bubbles.

### 2.3. Experiments to Determine the Influence of the Number of Windings of the Probe

The relationship between the number of windings of the probe to cell binding efficiency was analyzed. For these experiments probes with 0, 4, 8, 12, 16, 20, 24, and 32 windings were used. Each design was tested with at least four probes and a negative control. Due to the high number of probes, the experiment was divided into two parts and in total three flow systems. All experiments were performed using the flow system, which was expanded to fit the required number of probes. They were first blocked for 30 min using 3% BSA/PBS and subsequently filled with a dextran solution containing 2000 cells/mL. The probes were incubated for 60 min at room temperature and a volume flow rate of 907 mL/h.

The first studies contained probes with 4, 8, 12, and 16 windings. Each winding design was tested with seven probes and one negative control. Two flow systems were used, each containing four probes per windings configuration. The inserted probes were coated with anti-EpCAM ab (Miltenyi GmbH, Bergisch Gladbach, Germany) to target LNCaP cell culture cells (provided by Dr. Magdalena Mayer and Karol Marcinkowski, Medical University in Poznan, Poland). The second study contained the probes with 0, 16, 20, 24, and 32 windings. A BMProbe™ with 28 windings was not included due to limitations in the manufacturing process. For each number of windings, four probes were included along with two negative controls. All probes were included in one flow system that contained cell culture human umbilical vein endothelial cells (HUVEC) cells (Cat#1210111, Provitro GmbH, Berlin, Germany). The probes were coated with anti-CD105 ab (Miltenyi GmbH, Germany).

A different Ab-coating and cell line was used in the two studies, as the absolute number of bound cells between the two studies was not comparable to one another, due to the experiments being performed on different days. This time difference would have resulted in using different cell cultures anyway. The chosen experimental setup therefore also enabled testing the ability to coat the BMProbe™ with different Abs.

### 2.4. Staining Protocol

To quantify the cells bound to the BMProbe™, immunofluorescence (IF) imaging is performed. In preparation thereof, the fixed BMProbe™ is stained. If the BMProbe was stored dry at 4 °C (for cell culture cells) or −80 °C (for patient cells), this step can be performed up to three months after the cells were bound to the BMProbe™.

This study focuses on the application of the BMProbe™ to target CTCs in lung cancer patients. CTCs are defined as anti-EpCAM and anti-cytokeratin (CK) Ab positive. These markers are also relevant for CTCs derived from different epithelial cancers (e.g., in breast cancer [26], prostate cancer [27], and colorectal cancer [28]). Further, established systems like CellSearch also use anti-EpCAM and anti-CK Ab to identify CTCs in epithelial cancer patient blood analysis [29]. The CTCs are differentiated from possibly bound peripheral blood mononuclear cells (PBMC) by using the anti-CD45 Ab. While blood cells are CD45 positive, CTCs are CD45 negative. To confirm the accurate staining procedure a prepared positive control with blood cells and cell culture CTC is always included in the staining process.

**Figure 4:**
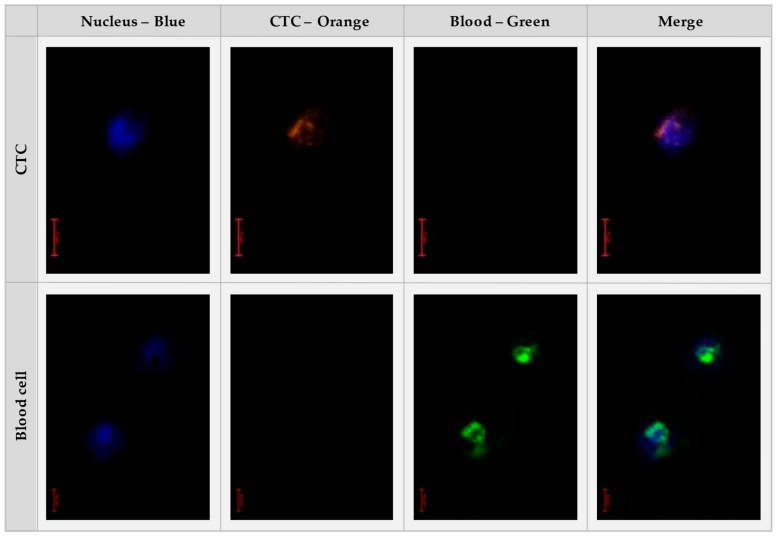
Staining images of a CTC (**top**) and blood cell (**bottom**). The cell nucleus is detected in the blue channel. CTCs are detected in the orange channel and blood cells are detected in the green channel. Finally, the images are superimposed to analyze the shape and position of the different spots.

In detail, this process runs as follows: Following a rehydration step of one minute in 450 µL of Phosphate Buffered Saline (PBS) (Carl RothGmbH + Co. KG, Karlsruhe, Germany) with a pH value of 7.4, the probes are blocked using a solution of 3% Bovine Serum Albumin (BSA, VWR Life Science, Radnor, PA, USA) in PBS for 30 min at 4 °C. 120 µL of multiplex staining solution is used for each BMProbe™. The staining solutions are prepared with 3% BSA in PBS at a dilution of 1:50 for anti-CD45-AlexaFluor488 (Exbio, Vestec, Czech Republic), 1:500 for anti-EpCAM-Phycoerythin (PE) (Miltenyi, Germany) and 1:250 for anti-CK-PE (Miltenyi, Germany) Abs. The BMProbes™ are then incubated in the dark capillaries, which protect the probes from light, these are filled with the staining dilution for 60 min at 4 °C and 200 rpm. This is followed by two washing steps, each for 1 min at 200 rpm. Then, the BMProbes™ are incubated for two minutes in a 500 µL Höchst (Thermo Fisher, Waltham, MA, USA) solution with a dilution of 1:10.000 diluted in PBS. Following three washing steps in PBS, each lasting for one minute, the BMProbe™ can be analyzed under the microscope. The staining process currently lasts 85 min in total. However, it is possible to stain many probes in parallel without increasing the total duration. Further, it is possible to store already stained probes at −80 °C for a maximum of one month until they are investigated. Figure 4 shows the microscopy images of the stained BMProbe™.

**Figure 5:**
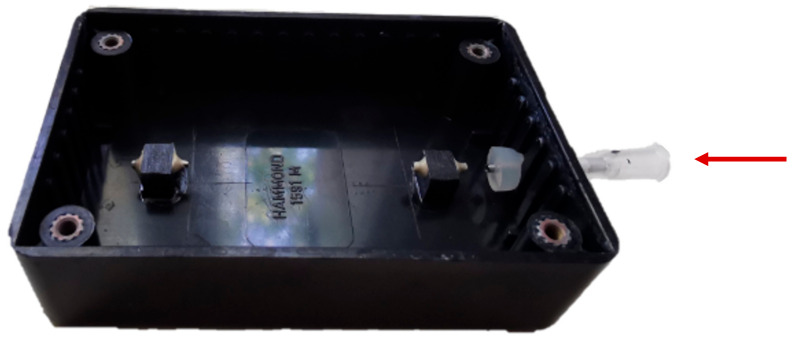
The wet chamber enables an analysis of the BMProbe™ under wet conditions. The BMProbe™ is held in a horizontal position. This supports a continuous analysis of the surface in focus. The black color absorbs the light so that there are no reflections. The BMProbe™ is inserted where the red arrow is pointing.

The microscopy was performed in-house using an Olympus BX51 microscope (Olympus, Tokio, Japan). For the analysis, the BMProbe™ is inserted through a Luer lock into the wet chamber that is filled with PBS. The wet chamber is a modified universal housing (Hammond Manufacturing Co., Ltd., Guelph, ON, Canada) that consists of two shortened G18 diameter cannulas that hold the BMProbe™ in a horizontal position so that it remains in focus during analysis. The wet chamber is made of black Acrylonitrile butadiene styrene to avoid reflection, it can be seen in Figure 5.

### 2.5. Validating the Functionality with Blood from Lung Cancer Patients

The following experiments were performed to validate the ex vivo functionality of the BMProbe™ and its platform technology approach. The study was conducted at the Lungenkrebszentrum of the Charité-Universitätsmedizin Berlin, Berlin, Germany. In this single-center, pseudonymized, and evaluator-blinded study, blood was withdrawn from healthy controls and non-small cell lung carcinoma (NSCLC) patients.

The experiments were performed in the previously shown flow system and the probes were analyzed according to the staining protocol introduced above. For each patient, blood was collected in 2 × 9 mL ethylene diamine tetraacetate (EDTA) vacuum tubes (Vacutest Kima srl, Arzergrande, Italy). First, 4 BMProbes™ were inserted into the flow system and then 10 mL of blood was inserted from the two EDTA vacuum tubes into the fluid reservoir of the flow system. Of the four BMProbes™, one was coated with anti-FOLR1 Ab (Biotechne Novus, Centennial, CO, USA) since Folate receptors are highly expressed in NSCLC [30,31,32]. Folate receptors can be found in the tissue of different organs, for example, lung, breast, and pancreas. In the circulatory system, only CTCs express folate receptors [33]. The second BMProbe™ was coated with anti-Vimentin Ab (Abnova, Taipei, Taiwan) to detect cells following the epithelial-mesenchymal transition. Xie et al. showed that cell surface vimentin positively correlated with lymph nodes and distant metastases in NSCLC [34]. The third was coated with the major known carcinoma cell surface protein anti-EpCAM Ab (Miltenyi GmbH, Germany), as it is the most frequently described cell surface marker for CTC enrichment in literature. It is used by established systems like CellSearch [35]. The fourth BMProbe™ was a negative control, that was not coated with any Abs. Previous experiments showed that the order in which the BMProbes™ are inserted into the flow system does not influence the binding efficiency.

Both cohorts were only recruited, if they were over the age of 18, neither pregnant nor breastfeeding, and had understood and signed the consent form. Healthy controls were included if they had no known prior history of cancer diseases. Due to their young age, no further tests were conducted to specifically rule out any unknown diseases/cancers. NSCLC patients (consented) were recruited that had been diagnosed with late-stage (stage III and IV) metastatic NSCLC and before treatment with chemotherapies or other therapies.

The aim of the experiments was to verify the functionality of the BMProbe™ by showing that more CTCs are isolated in the blood of lung cancer patients compared to in the blood of a healthy control group. The study did not intend to make any clinical prediction based on the number of isolated cells.

## 3. Results

### 3.1. Analysis of the Influence the Number of Windings Has on the Binding Efficiency

**Figure 6:**
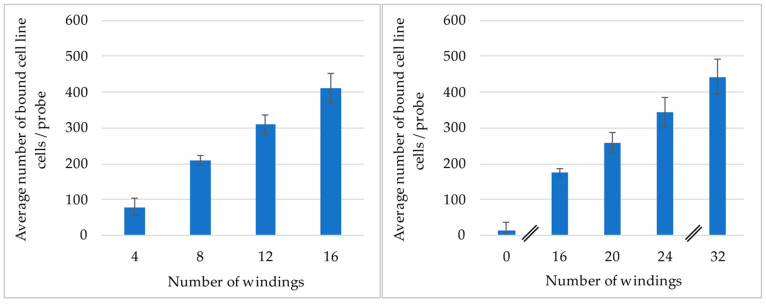
(**Left**), shows the cell binding efficiency using the LNCaP cell line and anti-EpCAM coating for the lower number of windings (N = 7). (**Right**), shows the cell binding efficiency for the HUVEC cell line and anti-CD 105 coating for the higher number of windings and for zero windings (N = 4).

The results of the two experiments are shown in Figure 6. The experiment with LNCaP prostate cancer cell line cells and anti-EpCAM coating regarding the windings 4, 8, 12, and 16 is shown on the left of Figure 6. There is a linear correlation between the number of windings and the cell binding efficiency (R^2^ = 0.9958). The mean relative standard deviation for each winding configuration is 13.7% (11.9–16.4%). The increase in bound cells between the windings 4 to 8, 8 to 12, and 12 to 16 is 130, 102, and 100, respectively. On the right of Figure 6, the binding efficiency of the probes with 0, 16, 20, 24, and 32 windings is shown. Here, the cardiovascular cell line HUVEC was used, see chapter 2.3 for explanation. The absolute number of bound cells on the probe is lower, compared to the first experiment. Nonetheless, there continues to be an increase in bound cells with an increase in windings (linear trend line, R^2^ = 0.9758). For the windings 16, 20, 24, and 32 the mean relative standard deviation for each winding configuration is 26.3% (21.7–29%). The probe with 0 windings has a relative standard deviation of 74.1%, which can be explained due to the low number of cells bound. Between the winding configurations 16 to 20, 20 to 24, and 24 to 32, the mean increase of bound cells is 85, 86, and 97, respectively.

### 3.2. Results of the Ex Vivo Experiments to Validate the Functionality of the BMProbe™

In total, 16 patients were recruited for this study, nine of which were healthy controls (four males and five females), who had a mean age of 29 years (±2.5 years). Seven metastatic NSCLC patients were recruited with a mean age of 72.9 years (±6.9 years).

**Figure 7:**
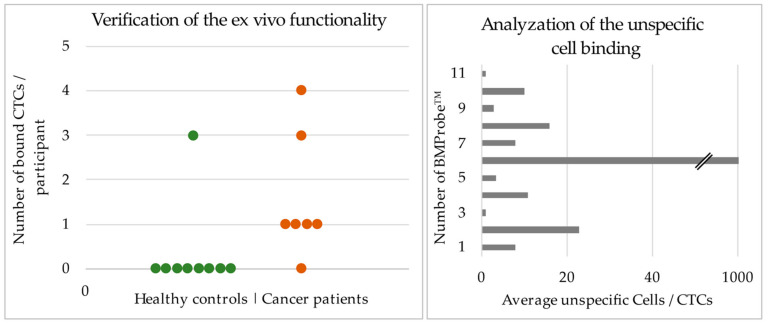
(**Left**), results from the ex vivo blood experiments using the flow system and BMProbes™. (**Right**), shows the ratio of unspecific cells to CTCs that are bound to the surface of the BMProbe™.

In Figure 7 (left) the cells bound to the three BMProbes™ coated with antibodies were added together for each study participant. A detailed Table showing the number of bound cells per BMProbe™ can be found in Supplementary Figure S1. The median number of bound cells to the surface of the BMProbes™ from the 10 mL blood sample is 0 (0–3) for the healthy control and 1 (0–4) for the cancer patients. Figure 7 (right) shows the ratio of unspecific binding to isolated CTCs. Only the BMProbes™ that had isolated at least one CTC were included. Therefore, Figure 7 (right) shows the number of bound nonspecific cells, divided by the number of bound lung cancer CTCs, for each BMProbe™. A median of 8 unspecific cells was isolated per CTC. The negative controls had 0 cells bound to the surface in all study participants.

If the cut-off is set at 0 CTCs to differentiate between healthy controls and lung cancer patients, it is possible to differentiate the two groups with a sensitivity of 85.7% and specificity of 88.9%.

## 4. Discussion

In this study, the relationship between the number of windings and cell binding efficiency was analyzed. Based on the experimental results it can be assumed that with an increase in windings, the flow is increasingly disrupted and redirected towards the wall. This leads to the 31-fold increase in cell binding efficiency between 32 windings and a flat wire indicating that the BMProbe™ should outperform the probes analyzed by Dizdar et al. [21] that create laminar flow conditions around the device. Therefore, it can be expected that in an in vivo application, multiple cells can be isolated.

The decrease of the cell binding gradient between 24 to 32 windings, compared to the gradient between 16 to 20 and 20 to 24, can possibly be explained through the gap between the windings. The smaller volume could start hindering the exchange of fluid passing in and out of the space between the windings. Theoretically, this could lead to a plateau of cell binding efficiency. However, due to manufacturing limitations, it was not possible to determine this plateau using experiments. Further, the analysis of the probes under the microscope for bound cells is expected to be more difficult with an increase in windings. The distance between the windings is large enough to easily analyze the entire area of each winding under the microscope for bound cells. With an increase in windings, this distance decreases, and the windings will become more perpendicular to the field of view. This makes it more difficult to screen the area of the windings for bound cells. The difference in the absolute number of bound cells between the experiments can be explained through the different coating and used cell line cells. It was not analyzed whether the cells bound to the surface of the probe were alive or dead. However, their ability to bind to the antibodies and their morphological profile during immunofluorescent staining suggest that the cells bound to the BMProbe™ are alive. For this study, it was only relevant that the cells were intact and therefore the cells can be alive or partially dead (undergoing apoptosis). An analysis of whether the cells are alive, or dead will be performed in the future.

For the ex vivo verification of the BMProbe™, the functionality to bind CTCs in lung cancer patient blood was analyzed in this study. NSCLC cancer was chosen as a clinically relevant application to validate the functionality, as it has been shown that CTCs have a prognostic and predictive value in NSCLC [36]. Despite advances in the treatment of lung cancer, the 2.09 million new lung cancer cases each year still cause nearly 1.8 million lung cancer-related deaths worldwide [37].

It is expected that the flow system for these experiments best mimicked the blood flow and cells to bind to the probe. While the flow system was developed to depict the median cubital vein (diameter and flow) in the area where the BMProbe™ is positioned, it has a couple of unphysiological characteristics that can potentially influence the CTCs. For example, the stopcocks that are used in the flow system to position the BMProbes™ have rigid small diameters that cause flow constrictions and that do not occur in the body. This can result in greater forces acting on the CTCs, especially through the jet streams and flow separation behind the hole from the stopcocks in the flow direction. Both characteristics could lead to cell damage. For the experiments performed with patient blood, this can explain why in the median, only one cell was bound to the surface of the BMProbe™. However, since the BMProbe™ is intended for the in vivo application, the cell loss caused by the flow system was not further analyzed. Should in future the BMProbe™ be used in an ex vivo setting, the blood volume during the blood draw should be increased. Using a greater blood volume has the potential for a greater cell yield.

Nonetheless, the results presented are very promising as they show that it is possible to isolate CTCs from the blood of NSCLC patients and that, in all but one case, no CTCs were isolated in the healthy controls. It should also be noted that the ex vivo results are from 10 mL of blood, which is a small amount and therefore only a low number of CTCs were expected. The actual application of the BMProbe™ will be in vivo where a far greater volume of blood will be screened and thus significantly more CTCs will be detected. Further, the low unspecific binding can lead to a more precise downstream analysis of the cells. The results also show the platform technology ability of the BMProbe™ as different antibodies were covalently bound to the surface and each antibody at one time or another successfully isolated CTCs. Further, the approach allows isolating and assessing CTCs from blood without drawing any amount of blood from the patient. Since the BMProbe™ can be coated with different Abs, potentially, CTCs can even be isolated from hematologic malignancies, if cancer-antigen-specific cell surface Abs are available [38].

The BMProbe™ intends to overcome the limitations of current ex vivo CTC isolation methods with its’ in vivo approach and flow-optimized geometry. Whereby a larger amount of peripheral blood is analyzed compared to the traditional 5 to 10 mL. It is expected that the CTC yield will be thereby increased, thus increasing the precision of downstream analysis. A reliable method for CTC isolation and analysis can be applied in the clinical setting for the purpose of diagnosis, monitoring, and decision support in the treatment of different types of cancer patients. Especially in cancers such as lung and prostate cancer that lack efficient methods for blood diagnosis. In Lung cancer, Zhao et al. summarized that CTCs are a promising biomarker to diagnose lung cancer with high sensitivity and specificity [39]. In prostate cancer, Ried et al. have shown that including a CTC analysis for the diagnosis of prostate cancer greatly increases the sensitivity and specificity of current diagnostic methods [40]. Regarding the translational aspect, the BMProbe™ has the potential to be integrated into a clinical diagnostic routine. The logistics are similar to a regular blood draw but without any blood draw thereby, other blood draw tests can be performed without large amounts of blood taken from patients, which can be a logistic problem in older or morbid patients. Furthermore, the ability to store the BMProbe™ between the patient application and analysis indicates that it does not have to be analyzed immediately on-site and can be shipped to a laboratory for later analysis. This also allows for the probe to be stored for weeks before analysis, which resolves an important logistic issue.

Currently, the counting of the bound cells to the surface of the BMProbe™ is performed using IF imaging. This is an easy-to-use method that uses relatively standard laboratory IF microscopy instrumentation. Cells can be quantified with specific imaging software. However, with the analytical IF microscopy instrumentation analysis today this is a very valuable approach, especially in combination with multiplex Ab assessment, quantification using imaging recognition and quantification. In the future, machine learning algorithms can be used to identify IF-stained cells and improve assessment with quantification software logistics. There are already different working groups that are developing and comparing machine learning algorithms that can identify CTCs (e.g., [41,42]). Integrating these algorithms in the analysis of the BMProbe™ would greatly reduce the hands-on duration of the counting of cells and reduce subjective errors by an individual reading the BMProbe™ bound CTCs

## 5. Conclusions

In this publication, the ex vivo functionality of the BMProbe™ was validated using 10 mL of lung cancer patient blood. The BMProbe™ is a minimally invasive probe that isolates the target cells directly from the peripheral venous bloodstream. This approach overcomes the limitations and problems associated with a blood draw and does not require withdrawing any amount of blood from patients. Further, the results presented in this study show that the geometry, more specifically the windings, of the BMProbe™ improve the cell deposition compared to a geometry that does not disrupt the flow close to the surface of the probe (e.g., a flat wire). The next step is to validate the in vivo functionality of the BMProbe™, where it is expected that multiple CTCs are isolated compared to ex vivo methods.

## Data Availability

The data presented in this study are available within the article and its Supplementary Data Files.

## Supplementary Materials

The following supporting information can be downloaded at: https://www.mdpi.com/article/10.3390/cancers14194753/s1. Figure S1: Thisdiagram shows the number of cells that were bound to each BMProbe™ in the NSCLC patient group and healthy control group. The BMProbes™ were coated with anti-FOLR1 Ab (left), anti-Vimentin Ab (middle) and anti-EpCAM Ab (right).

## References

[1] Turetta M., Bulfoni M., Brisotto G., Fasola G., Zanello A., Biscontin E., Mariuzzi L., Steffan A., Di Loreto C., Cesselli D. (2018). Assessment of the Mutational Status of NSCLC Using Hypermetabolic Circulating Tumor Cells. Cancers.

[2] Marchetti A., Del Grammastro M., Felicioni L., Malatesta S., Filice G., Centi I., De Pas T., Santoro A., Chella A., Brandes A.A. (2014). Assessment of EGFR Mutations in Circulating Tumor Cell Preparations from NSCLC Patients by Next Generation Sequencing: Toward a Real-Time Liquid Biopsy for Treatment. PLoS ONE.

[3] Markou A., Tzanikou E., Lianidou E. (2022). The Potential of Liquid Biopsy in the Management of Cancer Patients. Semin. Cancer Biol..

[4] Lozano R., Lorente D., Aragon I.M., Romero-Laorden N., Nombela P., Mateo J., Reid A.H.M., Cendón Y., Bianchini D., Llacer C. (2021). Value of Early Circulating Tumor Cells Dynamics to Estimate Docetaxel Benefit in Metastatic Castration-Resistant Prostate Cancer (MCRPC) Patients. Cancers.

[5] Jesus Magbanua M.M., Savenkov O., Asmus E.J., Ballman K.V., Scott J.H., Park J.W., Dickler M., Partridge A., Carey L., Winer E. (2020). Clinical Significance of Circulating Tumor Cells in Hormone Receptor-Positive Metastatic Breast Cancer Patients Who Received Letrozole with or without Bevacizumab HHS Public Access. Clin. Cancer Res..

[6] Hendricks A., Brandt B., Geisen R., Dall K., Röder C., Schafmayer C., Becker T., Hinz S., Sebens S. (2020). Isolation and Enumeration of CTC in Colorectal Cancer Patients: Introduction of a Novel Cell Imaging Approach and Comparison to Cellular and Molecular Detection Techniques. Cancers.

[7] Tay R.Y., Fernández-Gutiérrez F., Foy V., Burns K., Pierce J., Morris K., Priest L., Tugwood J., Ashcroft L., Lindsay C.R. (2019). Prognostic Value of Circulating Tumour Cells in Limited-Stage Small-Cell Lung Cancer: Analysis of the Concurrent Once-Daily versus Twice-Daily Radiotherapy (CONVERT) Randomised Controlled Trial. Ann. Oncol..

[8] Smit D.J., Pantel K., Jücker M. (2021). Circulating Tumor Cells as a Promising Target for Individualized Drug Susceptibility Tests in Cancer Therapy. Biochem. Pharmacol..

[9] Hosseini H., Obradović M., Hoffmann M., Harper K.L., Sosa M.S., Werner-Klein M., Nanduri L.K., Werno C., Ehrl C., Maneck M. (2016). Early Dissemination Seeds Metastasis in Breast Cancer. Nature.

[10] Yamaguchi J., Kokuryo T., Yokoyama Y., Ebata T., Ochiai Y., Nagino M. (2021). Premalignant Pancreatic Cells Seed Stealth Metastasis in Distant Organs in Mice. Oncogene.

[11] Neumann M.H.D., Bender S., Krahn T., Schlange T. (2018). CtDNA and CTCs in Liquid Biopsy—Current Status and Where We Need to Progress. Comput. Struct. Biotechnol. J..

[12] Chemi F., Mohan S., Guevara T., Clipson A., Rothwell D.G., Dive C. (2021). Early Dissemination of Circulating Tumor Cells: Biological and Clinical Insights. Front. Oncol..

[13] Alvarez Cubero M.J., Lorente J.A., Robles-Fernandez I., Rodriguez-Martinez A., Puche J.L., Serrano M.J. (2017). Circulating Tumor Cells: Markers and Methodologies for Enrichment and Detection. Methods Mol. Biol..

[14] Coumans F., van Dalum G., Terstappen L.W.M.M. (2018). CTC Technologies and Tools. Cytom. Part A.

[15] Ashworth T.R. (1869). A Case of Cancer in Which Cells Similar to Those in the Tumours Were Seen in the Blood after Death. Med. J. Aust..

[16] Lin D., Shen L., Luo M., Zhang K., Li J., Yang Q., Zhu F., Zhou D., Zheng S., Chen Y. (2021). Circulating Tumor Cells: Biology and Clinical Significance. Signal Transduct. Target. Ther..

[17] Yang Y.-P., Giret T.M., Cote R.J., Alix-Panabieres C., González Hernández Á. (2021). Cancers Circulating Tumor Cells from Enumeration to Analysis: Current Challenges and Future Opportunities. Cancers.

[18] Vermesh O., Aalipour A., Ge T.J., Saenz Y., Guo Y., Alam I.S., Park S.M., Adelson C.N., Mitsutake Y., Vilches-Moure J. (2018). An Intravascular Magnetic Wire for the High-Throughput Retrieval of Circulating Tumour Cells in Vivo. Nat. Biomed. Eng..

[19] Saucedo-Zeni N., Mewes S., Niestroj R., Gasiorowski L., Murawa D., Nowaczyk P., Tomasi T., Weber E., Dworacki G., Morgenthaler N.G. (2012). A Novel Method for the in Vivo Isolation of Circulating Tumor Cells from Peripheral Blood of Cancer Patients Using a Functionalized and Structured Medical Wire. Int. J. Oncol..

[20] Cieślikowski W.A., Budna-Tukan J., Świerczewska M., Ida A., Hrab M., Jankowiak A., Mazel M., Nowicki M., Milecki P., Pantel K. (2020). Circulating Tumor Cells as a Marker of Disseminated Disease in Patients with Newly Diagnosed High-Risk Prostate Cancer. Cancers.

[21] Dizdar L., Fluegen G., van Dalum G., Honisch E., Neves R.P., Niederacher D., Neubauer H., Fehm T., Rehders A., Krieg A. (2019). Detection of Circulating Tumor Cells in Colorectal Cancer Patients Using the GILUPI CellCollector: Results from a Prospective, Single-Center Study. Mol. Oncol..

[22] Hehnen F., Wendt G., Schaller J., Geus P., Villwock J., Kertzscher U., Goubergrits L. (2021). Investigation of the Attachment of Circulating Endothelial Cells to a Cell Probe: Combined Experimental and Numerical Study. Adv. Eng. Mater..

[23] Hofmann O., Voirin G., Niedermann P., Manz A. (2002). Three-Dimensional Microfluidic Confinement for Efficient Sample Delivery to Biosensor Surfaces. Application to Immunoassays on Planar Optical Waveguides. Anal. Chem..

[24] Spivack D.E., Kelly P., Gaughan J.P., Van Bemmelen P.S. (2012). Mapping of Superficial Extremity Veins: Normal Diameters and Trends in a Vascular Patient-Population. Ultrasound Med. Biol..

[25] Mukai K., Nakajima Y., Nakano T., Okuhira M., Kasashima A., Hayashi R., Yamashita M., Urai T., Nakatani T. (2020). Safety of Venipuncture Sites at the Cubital Fossa as Assessed by Ultrasonography. J. Patient Saf..

[26] Rossi G., Mu Z., Rademaker A.W., Austin L.K., Strickland K.S., Costa R.L.B., Nagy R.J., Zagonel V., Taxter T.J., Behdad A. (2018). Cell-Free DNA and Circulating Tumor Cells: Comprehensive Liquid Biopsy Analysis in Advanced Breast Cancer. Clin. Cancer Res..

[27] Goldkorn A., Ely B., Quinn D.I., Tangen C.M., Fink L.M., Xu T., Twardowski P., Van Veldhuizen P.J., Agarwal N., Carducci M.A. (2014). Circulating Tumor Cell Counts Are Prognostic of Overall Survival in SWOG S0421: A Phase III Trial of Docetaxel with or without Atrasentan for Metastatic Castration-Resistant Prostate Cancer. J. Clin. Oncol..

[28] Cohen S.J., Punt C.J.A., Iannotti N., Saidman B.H., Sabbath K.D., Gabrail N.Y., Picus J., Morse M.A., Mitchell E., Miller M.C. (2009). Prognostic Significance of Circulating Tumor Cells in Patients with Metastatic Colorectal Cancer. Ann. Oncol..

[29] De Wit S., Van Dalum G., Lenferink A.T.M., Tibbe A.G.J., Hiltermann T.J.N., Groen H.J.M., Van Rijn C.J.M., Terstappen L.W.M.M. (2015). The Detection of EpCAM+ and EpCAM- Circulating Tumor Cells. Sci. Rep..

[30] O’Shannessy D.J., Yu G., Smale R., Fu Y.S., Singhal S., Thiel R.P., Somers E.B., Vachani A. (2012). Folate Receptor Alpha Expression in Lung Cancer: Diagnostic and Prognostic Significance. Oncotarget.

[31] Nunez M.I., Behrens C., Woods D.M., Lin H., Suraokar M., Kadara H., Hofstetter W., Kalhor N., Lee J.J., Franklin W. (2012). High Expression of Folate Receptor Alpha in Lung Cancer Correlates with Adenocarcinoma Histology and EGFR [Corrected] Mutation. J. Thorac. Oncol..

[32] Chen X., Zhou F., Li X., Yang G., Zhang L., Ren S., Zhao C., Deng Q., Li W., Gao G. (2015). Folate Receptor-Positive Circulating Tumor Cell Detected by LT-PCR-Based Method as a Diagnostic Biomarker for Non-Small-Cell Lung Cancer. J. Thorac. Oncol..

[33] Parker N., Turk M.J., Westrick E., Lewis J.D., Low P.S., Leamon C.P. (2005). Folate Receptor Expression in Carcinomas and Normal Tissues Determined by a Quantitative Radioligand Binding Assay. Anal. Biochem..

[34] Xie X., Wang L., Wang X., Fan W.H., Qin Y., Lin X., Xie Z., Liu M., Ouyang M., Li S. (2021). Evaluation of Cell Surface Vimentin Positive Circulating Tumor Cells as a Diagnostic Biomarker for Lung Cancer. Front. Oncol..

[35] Gerratana L., Davis A.A., Polano M., Zhang Q., Shah A.N., Lin C., Basile D., Toffoli G., Wehbe F., Puglisi F. (2021). Understanding the Organ Tropism of Metastatic Breast Cancer through the Combination of Liquid Biopsy Tools. Eur. J. Cancer.

[36] Tamminga M., De Wit S., Schuuring E., Timens W., Terstappen L.W.M.M., Hiltermann T.J.N., Groen H.J.M. (2019). Circulating Tumor Cells in Lung Cancer Are Prognostic and Predictive for Worse Tumor Response in Both Targeted- and Chemotherapy. Transl. Lung Cancer Res..

[37] Ferlay J., Colombet M., Soerjomataram I., Parkin D.M., Piñeros M., Znaor A., Bray F. (2021). Cancer Statistics for the Year 2020: An Overview. Int. J. Cancer.

[38] Abdulmawjood B., Roma-Rodrigues C., Fernandes A.R., Baptista P.V. (2019). Liquid Biopsies in Myeloid Malignancies. Cancer Drug Resist..

[39] Zhao Q., Yuan Z., Wang H., Zhang H., Duan G., Zhang X. (2021). Role of Circulating Tumor Cells in Diagnosis of Lung Cancer: A Systematic Review and Meta-Analysis. J. Int. Med. Res..

[40] Ried K., Tamanna T., Matthews S., Eng P., Sali A. (2020). New Screening Test Improves Detection of Prostate Cancer Using Circulating Tumor Cells and Prostate-Specific Markers. Front. Oncol..

[41] Stevens M., Nanou A., Terstappen L.W.M.M., Driemel C., Stoecklein N.H., Coumans F.A.W. (2022). StarDist Image Segmentation Improves Circulating Tumor Cell Detection. Cancers.

[42] He B., Lu Q., Lang J., Yu H., Peng C., Bing P., Li S., Zhou Q., Liang Y., Tian G. (2020). A New Method for CTC Images Recognition Based on Machine Learning. Front. Bioeng. Biotechnol..

