# Supplementary figures and images for: Verification of a Novel Minimally Invasive Device for the Isolation of Rare Circulating Tumor Cells (CTC) in Cancer Patients’ Blood
Source: Cancers (Basel). 2022 Sep 29;14(19):4753. doi: 10.3390/cancers14194753 (PMC9562020; doi:10.3390/cancers14194753)

Supplementary figure S1

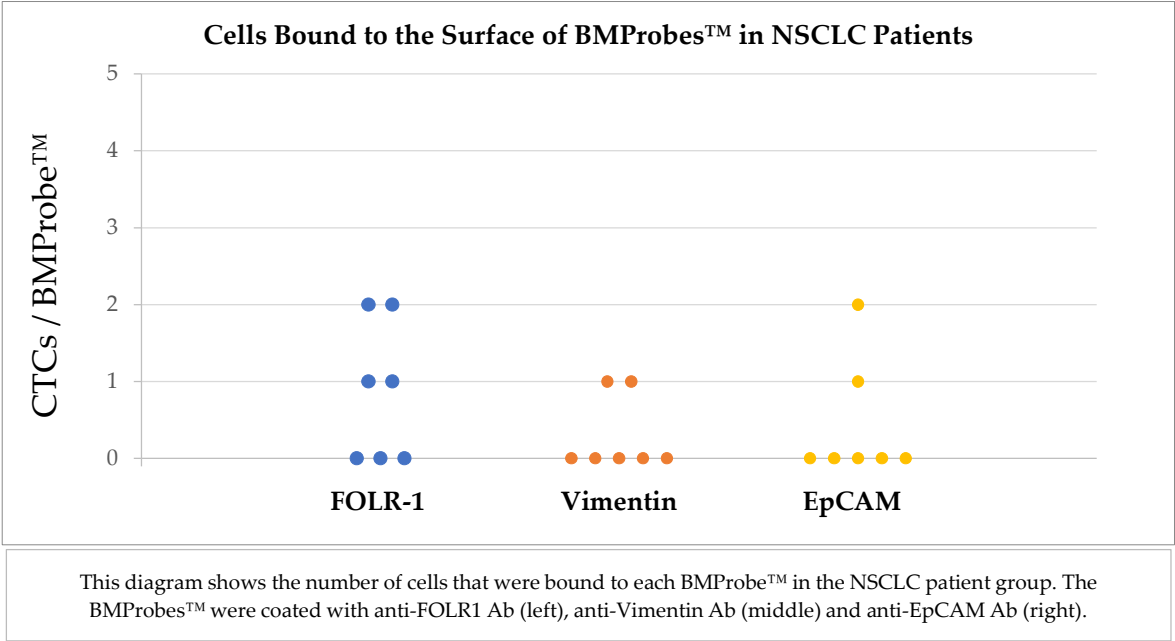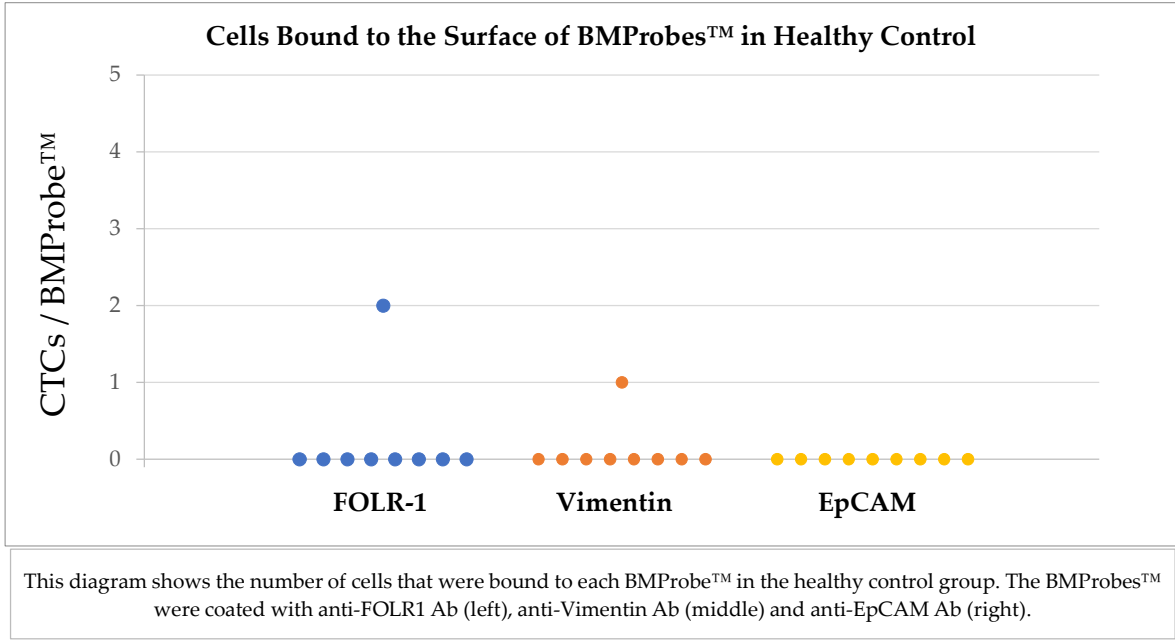

Supplement: Supplementary file 1 [file cancers-14-04753-s001.zip › cancers-1947534-supplementary.pdf]
